# Quantitative Changes in the Proteome of Chronically Inflamed Lacrimal Glands From a Sjögren's Disease Animal Model

**DOI:** 10.1167/iovs.66.4.44

**Published:** 2025-04-17

**Authors:** Danny Toribio, Junji Morokuma, Dante Pellino, Markus Hardt, Driss Zoukhri

**Affiliations:** 1 Department of Basic and Clinical Translational Sciences, Tufts University School of Dental Medicine, Boston, Massachusetts, United States; 2 Center for Salivary Diagnostics, ADA Forsyth Institute, Cambridge, Massachusetts, United States; 3 Department of Inflammation and Immunology, ADA Forsyth Institute, Cambridge, Massachusetts, United States; 4 Department of Ophthalmology, Tufts University School of Medicine, Boston, Massachusetts, United States

**Keywords:** lacrimal gland (LG), dry eye disease, Sjögren's disease, comparative proteomics, mass spectrometry, inflammation

## Abstract

**Purpose:**

The lacrimal gland (LG) is the major source of aqueous tears, and insufficient LG secretion leads to aqueous-deficient dry eye (ADDE) disease. To provide a foundational description of LG’s protein expression patterns, we prepared protein extracts of LGs from a wild-type and an ADDE mouse model and analyzed the proteome by quantitative mass spectrometry.

**Methods:**

LGs were isolated from an ADDE mouse model, male non-obese diabetic (NOD) mice and control wild-type BALB/c mice (*n* = 6 each). Protein samples were prepared in urea-based lysis buffer and protein concentrations determined by the BCA method. The equivalent of 200 µg protein were tryptically digested and analyzed by nanoflow liquid chromatography tandem mass spectrometry (LC-MS/MS). Proteins were identified and quantified using the PEAKS X bioinformatics suite. Downstream differential protein expression analysis was performed using the MS-DAP R package. Selected significantly differentially expressed and detected proteins were subjected to spatial expression analysis using immunohistochemistry.

**Results:**

Cumulatively, the LC-MS/MS-based proteomics analyses of the murine LG samples identified a total of 31,932 peptide sequences resulting in 2617 protein identifications at a 1% false discovery rate at the peptide and protein level. Principal component analysis (PCA) and hierarchical cluster analysis revealed a separation of NOD and BALB/c samples. Overall, protein diversity was consistently higher in NOD samples. After applying global peptide filter criteria and peptide-to-protein rollup, 1750 remaining proteins were subjected to differential expression analysis using the MSqRob algorithm, which identified 580 proteins with statistically significant expression differences. Data are available via ProteomeXchange with identifier PXD060937. At the cellular level, the up- and downregulation of select proteins were confirmed by immunohistochemistry.

**Conclusions:**

Our data suggest that chronic inflammation leads to significant alterations in the LG proteome. Ongoing studies aim to identify potentially unique, inflammation-induced proteins that could be amenable to pharmacological modulation.

The lacrimal gland (LG) is responsible for providing the major components of the aqueous layer composing the human tear film.^1^ Secretions from the LG provide essential proteins, growth factors, anti-microbial agents, electrolytes, and immune cells required to nourish, protect, and help regulate the function of the cornea and conjunctiva.^1^^–^^3^ Dysfunction of the LG can be induced by inflammation, aging, and other biological and/or environmental factors, resulting in insufficient lacrimal fluid and tear production.^1^^,^^4^ Insufficient tear production and low-quality lacrimal fluid leads to aqueous-deficient dry eye (ADDE) disease.^4^ Patients with ADDE disease often experience severe pain caused by irritation of the eye, blurry vision, and sensitivity to light. Moreover, severe dry eye disease can cause corneal ulcers and loss of corneal transparency, threatening vision and deteriorating the daily quality of life.^3^ ADDE has been recently subclassified into three types: type I (sporadic/intermittent disease), type II (persistent disease and the presence of an inflammatory component), and type III (chronic disease), based on the ability of the ocular surface to restore its equilibrium following exposure to key triggering factors, such as hyperosmolarity and inflammation.^5^

Sjögren's disease,^6^ the leading cause of ADDE, is a chronic autoimmune disorder of the lacrimal and salivary glands affecting an estimated 1 to 4 million Americans (mostly women) that results in dry eye and dry mouth.^7^ Sjögren's disease can be clinically manifested as a primary disorder (primary Sjögren's disease) or be associated with other autoimmune diseases (secondary Sjögren's disease), such as rheumatoid arthritis or systemic sclerosis.^4^ Sjögren's disease is characterized by focal lymphocytic infiltration of the lacrimal and salivary glands, and the production of autoantibodies, such as those directed against the ribonucleoproteins Ro/SSA and La/SSB.^4^ Additionally, an increased expression of several proinflammatory cytokines is commonly observed in the lacrimal and salivary glands of patients with Sjögren's disease.^4^ Many of these proinflammatory cytokines, as we and others have previously reported, impair LG myoepithelial cells contractile function, and inhibit neurally stimulated LG secretion.^8^^,^^9^

Male non-obese diabetic (NOD) mice have been extensively used as an animal model for human primary Sjögren's disease.^10^^–^^12^ In these mice, lymphocytic infiltration of the LG and disease severity occur in a time-dependent manner with older (>30 weeks) mice showing the most severe phenotype.^13^ Wild type age- and sex-matched BALB/c mice are an accepted control strain to use along the NOD mice as they do not showcase signs of inflammation.^14^

An inflammatory proteomic profile has been reported to be present in the tear film of patients with Sjögren's disease when compared with healthy controls.^1^ Several comparative studies have described the use of proteomic techniques to analyze and characterize the tear and salivary proteomes of healthy patients and patients with Sjögren's disease or murine models of the disease.^15^^–^^18^ Nonetheless, and to our knowledge, most of these proteomic-based studies have focused on lacrimal fluid, tear fluid, and/or saliva samples, and not on the proteome of chronically inflamed and diseased glands themselves. Despite all the efforts and research to understand the pathophysiology, molecular markers, and general factors involved in ADDE disease, the underlying mechanisms causing insufficient LG secretion due to chronic inflammation are still not well understood, and, thus, effective treatment modalities are still lacking.^4^^,^^19^

Proteomics techniques provide powerful tools enabling the study of proteins at a large scale. Liquid chromatography tandem mass spectrometry (LC-MS/MS) has now been, for over more than a decade, the main crucial tool in proteomics research.^20^ In recent years, LC-MS/MS has been increasingly applied in the identification of disease biomarkers and analysis of therapeutic drug monitoring, enabling the optimization of drug treatments for patients.^21^ Therefore, the aim of the present study was to (1) provide a comprehensive overview of the LG proteome alterations induced by chronic inflammation as occurs in ADDE disease and Sjögren's disease, and (2) identify potential novel protein-based biomarkers that could be used for future dry eye therapies through pharmacological modulation.

## Materials and Methods

**Figure 1.**
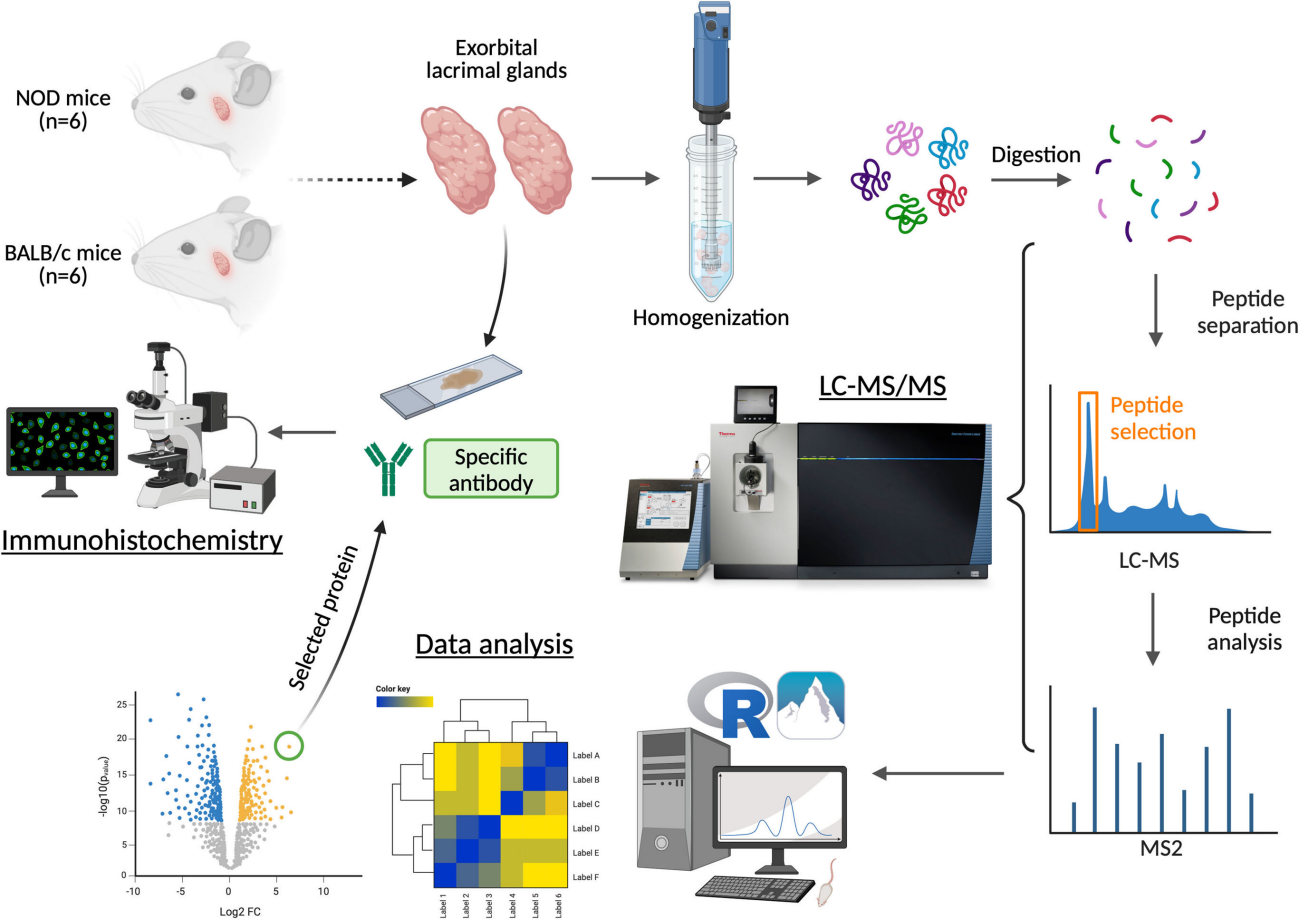
**Schematic overview of the study workflow.** Lacrimal glands (LGs) were isolated from a Sjögren's disease mouse model, 38-week-old male NOR/LtJ, an insulitis resistant and diabetes-free strain derived from non-obese diabetic (NOD) mice, and isolated from their respective sex- and age-matched wild type control, BALB/c mice (*n* = 6 each). Protein samples were prepared in 8M urea-based lysis buffer and protein concentrations determined by the BCA method. The equivalent of 200 µg protein were tryptically digested and analyzed by nanoflow liquid chromatography tandem mass spectrometry (LC-MS/MS). Proteins were identified and quantified using the PEAKS X bioinformatics suite. Downstream differential protein expression analysis was performed using the MS-DAP R software package. Selected significantly differentially expressed/detected proteins were subjected to spatial expression analysis using immunohistochemistry. Figure created with BioRender.com.

An overview of the study workflow is presented schematically in Figure 1. In this study, using algorithmic statistical models for differential expression analysis (DEA), the protein expression patterns of LGs from NOD mice, an ADDE, and excellent Sjögren's disease animal model, were compared with those from BALB/c mice – control animals that do not show signs of inflammation^14^ (Supplementary Fig. S1).

### Animals

All procedures were performed in accordance with the Association for Research in Vision and Ophthalmology (ARVO) statement for the use of animals in ophthalmic and vision research and were approved by the Tufts Medical Center Institutional Animal Care and Use Committee. Thirty-eight-week-old, male NOR/LtJ mice (catalog # 002050, purchased from The Jackson Laboratory, Bar Harbor, ME, USA) were used in this study. NOR/LtJ mice are an insulitis resistant and diabetes-free strain that still develops peripheral T-lymphocyte accumulation as characterized in NOD/ShiLtJ mice.^22^ Their respective sex- and age-matched controls, BALB/c mice (catalog # 028) were purchased from Charles River Laboratories (Wilmington, MA, USA). Animals were euthanized and their exorbital LGs excised and processed for protein extraction or histopathological procedures, as described below.

### Protein Digestion and Peptide Purification

Frozen LGs from NOD (*n* = 6) and BALB/c (*n* = 6) mice were resuspended in lysis buffer (8 M urea, 75 mM NaCl, 50 mM Tris-HCl [pH 8], 1 mM NaF, 1 mM β-glycerophosphate, 1 mM sodium orthovanadate, 10 mM sodium pyrophosphate, 1 mM PMSF, and 1 tablet of EDTA-free protease inhibitor cocktail) and homogenized using sonication. Homogenates were clarified by centrifugation at 16,000 × *g* for 10 minutes at 4°C and protein concentrations were determined using the bicinchoninic acid (BCA) assay method.

The equivalent of 200 µg of protein were subjected to protein digestion following a modified S-Trap protocol. Briefly, protein extracts were supplemented to a final concentration of 5% SDS and 50 mM triethylammonium bicarbonate (TEAB) prior to reduction with 5 mM tris(2-carboxyethyl) phosphine (TCEP) for 15 minutes at 56°C. Samples were cooled to room temperature (RT) and alkylated with 20 mM methyl methanethiosulfonate (MMTS) for 10 minutes. Lysates were acidified to a final concentration of 1.1% phosphoric acid, mixed with binding/wash buffer (BW = 90% methanol, and 100 mM TEAB) and the resulting protein suspension was transferred to the S-Trap and centrifuged at 2800 × *g* for 1 minute. Trapped proteins were washed four times with BW buffer. Then, 5 µg Trypsin/Lys-C (in 125 µL 50 mM TEAB) were added to each sample and incubated overnight at 37°C. The tryptic peptides were eluted via centrifugation for 1 minute at 2800 × *g* sequentially with 80 µL of TEAB, 80 µL of 0.2% TFA, and 80 µL of 50% ACN in 0.2% TFA. The eluted peptides were vacuum-dried and resuspended in 5% ACN in 0.2% TFA.

### LC-MS/MS Analysis

Nanoflow LC-MS/MS measurements were performed using an Easy-nLC 1000 system coupled to an Orbitrap Fusion mass spectrometer (Thermo Fisher Scientific, Waltham, MA, USA) for label-free quantitation. Tryptic peptides were trapped using an Acclaim PepMap 100 (100 µm × 2 cm) column, washed in 0.1% formic acid, and transferred to an Easy-Spray C18 (ES900, 75 µm × 15 cm, 3 µm particle size) analytical column (Thermo Fisher Scientific, Waltham, MA, USA) at a flow rate of 300 nL/min and a column temperature of 45°C. Chromatographic separation was achieved using a linear gradient up to 44% solvent B (80% acetonitrile and 0.1% (v/v) formic acid) over 120 minutes.

The mass spectrometer (MS) was operated in data-depended acquisition mode to automatically switch between MS and MS/MS measurements. Full scan MS spectra were recorded in the Orbitrap in the scan range from 350 to 1200 m/z at a resolution of 120,000 using an automatic gain control (AGC) target of 3.0 × 105 ion counts and a maximum injection time (maxIT) of 250 ms. After the survey scan, the 20 most abundant precursor ions (intensity ≥ 2.5 × 104 ions) were isolated in the quadrupole with an isolation window of a 1.6 m/z for high-collision dissociation fragmentation and fragment ions were recorded in the Orbitrap at a 15,000 resolution, AGC and maxIT were set to 5 × 104 and 150 ms, respectively. The dynamic exclusion time for previously analyzed precursor ions and isotopes was 40 seconds.

### Identification and Quantification of Peptides and Proteins

Raw MS data were processed using PEAKS Studio XPro 10.6 software using the following parameters: merge scans (retention time window = 10 ppm, precursor m/z tolerance = 10 ppm, and precursor mass and charge states z = 1–10). Other data pre-processing (centroiding, deisotoping, and deconvolution) was executed automatically. Searches were performed iteratively against the house mouse (*Mus* *Musculus*) UniProt database (July 2021, containing 15,000 entries) and the Mouse Oral Microbiome Database (MOMD; February 2023, containing 148,242 entries) using the following search parameters: parent mass error tolerance 10 ppm; fragment mass error tolerance 0.02 Da; trypsin enzyme specificity with cleavage prior to proline permitted; variable modifications: Methylthio (C), phosphorylation (STY), pyro-glu (Q), oxidation (M), deamidation (NQ), HexNAcylation (N), acetylation (N-term); one non-specific cleavage specificity on one terminus, maximal two missed cleavages, and maximal two variable post-translational modifications per peptide. Peptide-spectrum matches (PSMs) and proteins were filtered at a false discovery rate of 1% calculated using the decoy-fusion approach. Proteins were considered identified with at least two unique peptides. For protein quantification, the peak areas of all identified peak features were exported and further bioinformatics analysis were carried out in R software (version 4.4.01) using the MS-DAP 1.1.2 package.^23^^,^^24^ Specifically, peptides were globally filtered (quantified in at least three samples; at least two peptides per protein) prior to normalization using a combination of the robust linear regression (rlr) algorithm and subsequent balancing of between-group protein level fold changes with mode-between protein normalization.^25^ Subsequentially, peptide-to-protein-rollup was performed using the MaxLFQ-algorithm and DEA using the MSqRob algorithm.^26^ The MS-DAP differential detection algorithm was used to rank and prioritize proteins-of-interest not evaluated by the DEA algorithm due to insufficient data points across the entire dataset and a strong difference between the number of detected underlying peptides between the two sample groups.

### Gene Set Enrichment Analysis

The (fgsea) R software package was used for gene set enrichment analysis (GSEA) using all quantified proteins and a combination of their effect sizes and differential expression significance (adjusted *P* values) as a pre-ranking metric. Effect sizes indicate how meaningful the difference between the two groups is, allowing us to provide not only a statistical significance (*P* value), but also a substantive significance for how large the protein expression differences between NOD and BALB/c samples are.^27^ GSEA was performed against the M2, M5, and M8 collections of the Molecular Signatures database (MSigDB version 2023.1.Mm updated March 2023) that contain curated gene sets from online pathway databases, as well as ontology and cell type signature gene sets, respectively.

### Immunofluorescence Staining

LGs from at least 3 different NOD and BALB/c mice were fixed in 4% methanol-free formaldehyde diluted in PBS. Glands were washed in PBS and dehydrated using a series of xylenes and graded ethanol solutions prior to embedding. LG tissue was then embedded into paraffin blocks and 7-µm-thick tissue sections were cut and mounted onto Superfrost Plus microscope slides (Thermo Fisher Scientific, Waltham, MA, USA). Tissue sections were then dried in an Isotemp oven at 55°C for 15 minutes, deparaffinized in 3 washes of xylenes, and rehydrated using a series of gradient ethanol solutions, and a final wash in deionized/pure water. Next, antigen unmasking was performed by placing the slides in either an EDTA buffer (1 mM EDTA, 0.05% Tween 20, and pH 8.0) or citric-based antigen retrieval solution (pH 6.0; BD Biosciences, Franklin Lakes, NJ, USA) prior to microwaving them for 5 minutes. Tissue sections were allowed to cool down at RT for 20 minutes, and, when appropriate, tissue permeabilization was performed by incubation with 0.1% Triton X-100 in 1% BSA/PBS at RT for 10 minutes. After 3 washes with TBS, non-specific binding sites were then blocked using 10% normal donkey serum (Jackson Immuno Research Laboratories, West Grove, PA, USA) for 1 hour at RT. Tissue sections were then left incubating overnight, at 4°C, with the appropriate primary antibody (see Supplementary Table S1 for a list of antibodies used). Primary antibody was omitted for sections used as negative controls (Supplementary Fig. S2). Tissue sections were then incubated with a Donkey anti-Rabbit IgG H&L, Alexa Fluor 488 conjugated secondary antibody (1:1000 dilution; Abcam, Waltham, MA, USA) for 1 hour at RT. Any endogenous tissue fluorescence was then quenched using Invitrogen's Ready Probes Tissue Autofluorescence Quenching kit (Thermo Fisher Scientific, Waltham, MA, USA) prior to covering the slides with a SlowFade Diamond Antifade mounting medium (Thermo Fisher Scientific, Waltham, MA, USA) containing DAPI for nuclear counterstaining.

For double staining immunofluorescence experiments, LG tissue sections were processed as described above and incubated with directly labeled primary antibodies. Rabbit primary antibodies were conjugated using either the FlexAble 2.0 CoraLite Plus 488 or FlexAble 2.0 CoraLite Plus 555 antibody labeling kits for rabbit IgG as instructed by the manufacturer (Proteintech, Rosemont, IL, USA). Briefly, each antibody was labeled individually in a separate microtube with the corresponding fluorochrome and later mixed for the desired application. After deparaffinization, rehydration, antigen retrieval, and tissue permeabilization, tissue sections were blocked for 45 minutes using 1% bovine serum albumin and incubated overnight at 4°C with the labeled antibodies mixture (see Supplementary Table S1 for a list of antibodies used). Then, tissue sections were washed 3 times with TBST (0.05% Tween-20) and quenched for autofluorescence prior to cover slipping as described above.

### Immunofluorescence Image Acquisition and Analysis

Immuno-stained slides were imaged using a color digital camera (SPOT Insight CMOS; SPOT Imaging, Sterling Heights, MI, USA) mounted on an Eclipse E600 microscope (Nikon Instruments Inc., Melville, NY, USA). Two to three images (from 3 serial tissue sections on each slide) were randomly captured using the same exposure and gain parameters between BALB/c and NOD tissue sections. As described on ImageJ's webpage,^28^ the color threshold tool on the FIJI software (ImageJ version 1.54, National Institute of Health, Bethesda, MD, USA) was used to manually select the positively stained areas of each image. Subsequently, the mean gray value calculated for each selected area per image was used as a measurement of fluorescence intensity.^29^

Statistical analyses of immunofluorescence images and data plots were made using the GraphPad Prism Software (version 10.2.3; Boston, MA, USA). Where appropriate, data are presented as means ± standard deviation (SD). Any outliers were identified and removed using the ROUT method (Q = 1%) and normal data distribution was evaluated using normality and lognormality tests. Data were analyzed using two-tailed unpaired *t-*test with Welch's correction. Statistically significant differences between each group were considered at *P* value < 0.05.

## Results

### LC-MS/MS Identification of Peptides and Proteins

**Figure 2.**
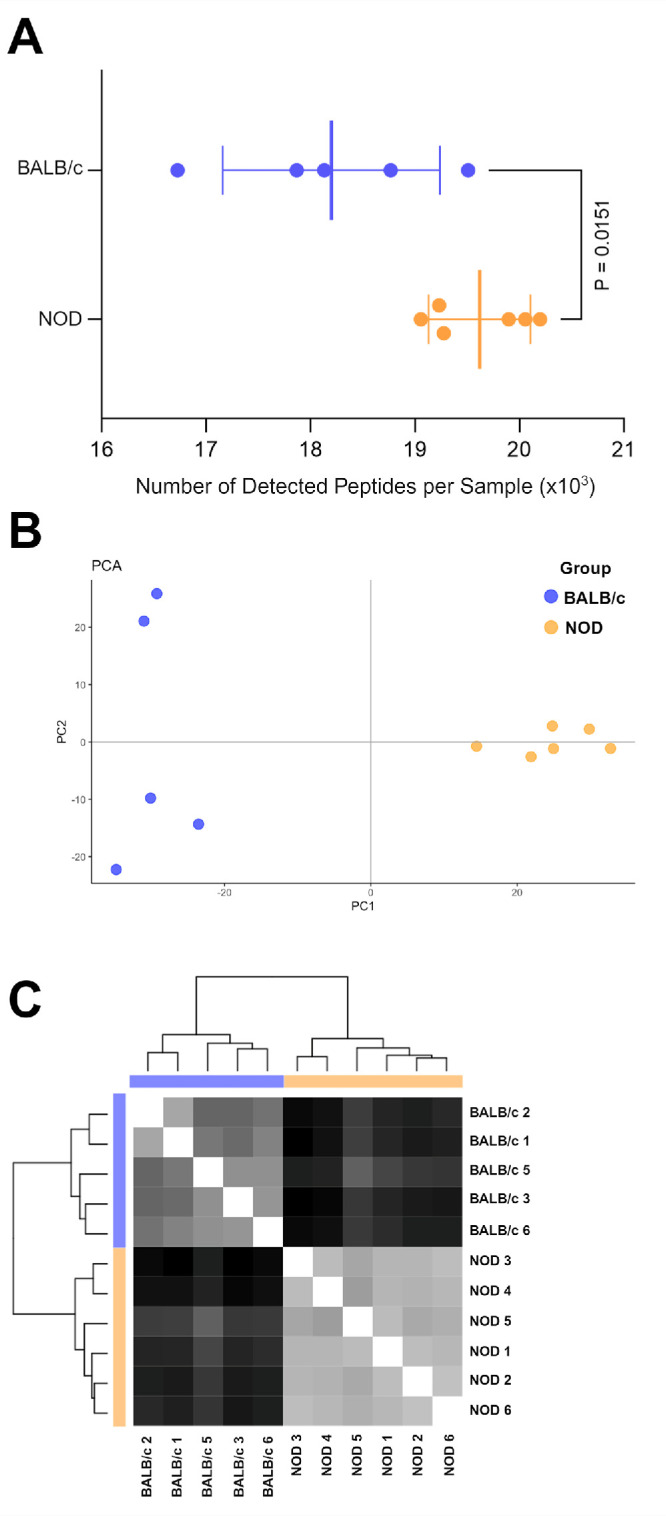
**Identification of peptides and proteins using LC-MS/MS.** Raw mass spectrometry data were processed using the PEAKS Studio XPro 10.6 software and searches were performed against the *M. musculus* UniProt and the Mouse Oral Microbiome (MOMD) databases, as described in the Materials and Methods section*.* (**A**) Data plot showing the number of identified peptides per sample for each group (18,200 ± 1000 detected peptides in BALB/c samples compared with 19,620 ± 500 in NOD samples, 2-tailed unpaired *t*-test). (**B**) Principal component analysis (PCA) plot showing clear separation of LG samples from each group (*blue dots* depict BALB/c samples whereas *orange dots* indicate NOD samples). (**C**) Hierarchical cluster analysis depicting a consistent group-specific clustering of BALB/c and NOD samples.

A total of 31,932 peptide sequences were identified by nanoflow LC-MS/MS resulting in 2617 protein identifications at a 1% false discovery rate at the peptide and protein level (the complete list of identified proteins is provided in Supplementary Table S2). The number of identified peptides per sample was, on average, statistically significantly lower in the control BALB/c samples when compared with NOD samples and showed higher levels of in-group variance (18,200 ± 1000 compared with 19,620 ± 500 peptides in BALB/c and NOD samples, respectively, two-tailed unpaired *t*-test; Fig. 2A). Quality control (QC) analysis revealed technical issues with sample “BALB/c 4” which was excluded from further analysis. Principal component analysis (PCA) showed a clear separation of LG samples from diseased NOD and control BALB/c mice (Fig. 2B). Similarly, a hierarchical cluster analysis revealed a consistent group specific clustering of BALB/c and NOD samples (Fig. 2C).

### Differential Expression Analysis and Differentially Detected Proteins

**Figure 3.**
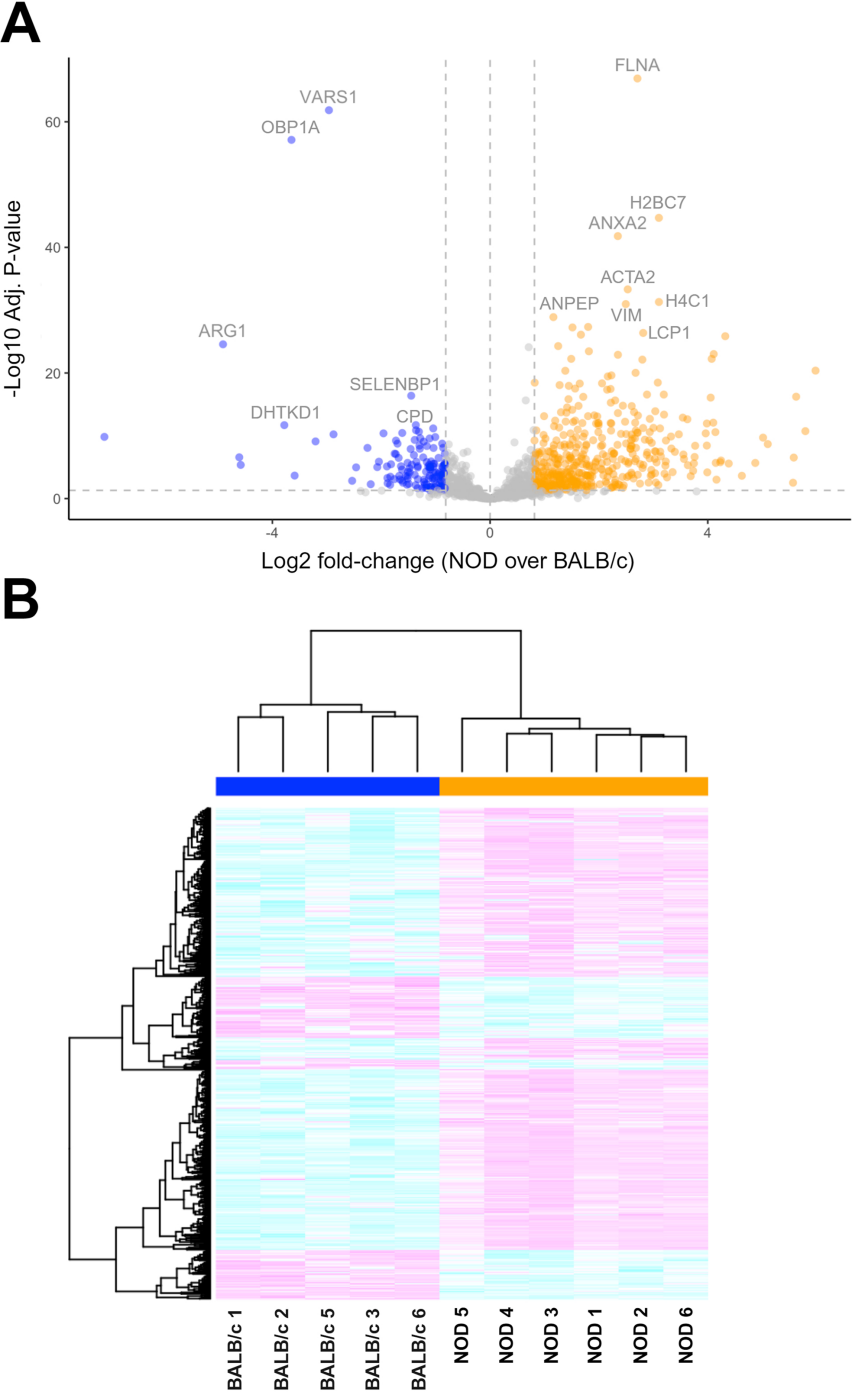
**Differentially expressed and detected proteins.** After global peptide filtering and peptide-to-protein-rollup, as described in the Materials and Methods section, the remaining proteins were subjected to differential expression analysis (DEA) using the MSqRob algorithm. (**A**) Volcano plot of differentially expressed proteins in LGs from diseased NOD mice compared with control BALB/c mice (*q*-value and bootstrapped fold-change cutoffs were defined to be 0.05 and 0.815, respectively). Some of the top 10 most significantly up- and downregulated proteins are specifically labeled. Proteins are ranked in the volcano plot based on their statistical significance (−log_10_ adjusted *P* values; y-axis) and their relative expression ratio between NOD and BALB/c samples (log_2_ fold-changes; x-axis). A positive log_2_ fold-change value (*orange spots*) indicates that protein abundance is higher in NOD samples and a negative fold-change (*blue spots*) indicates the exact opposite. (**B**) Heatmap of combined differentially expressed and differentially detected proteins corroborating the group-specific and distinctive protein expression profiles of NOD versus BALB/c clusters.

After applying the global peptide filtering criteria and peptide-to-protein-rollup, the remaining 1750 proteins were subjected to DEA using the MSqRob algorithm. DEA analyses identified 580 proteins that had statistically significant expression differences between BALB/c and NOD samples after correction for multiple comparisons (see Supplementary Table S3 for all 580 proteins with significant expression differences). To optimally visualize and display the DEA results, a volcano plot with defined q-value and foldchange cutoffs (q-value threshold = 0.05; bootstrapped log2 fold change threshold = 0.815) was created (Fig. 3A). A heatmap analysis of the differentially expressed and differentially detected proteins confirmed the distinct protein expression profiles of BALB/c versus NOD clusters (Fig. 3B).

We took into consideration both the log2 fold changes and differential expression significance (adjusted *P* values) of all differentially expressed and differentially detected proteins as a ranking criterion to identify the most significantly up- and downregulated proteins in our dataset. This ranking method allow us to take all proteins with significant expression changes into consideration and not only those that have large expression changes (such as fold changes ≥ 2) as it is frequently done. The top 10 most significantly up- and downregulated proteins in the LGs of diseased NOD mice compared with control mice are presented separately in Tables 1 and 2.

**Table 1.** Top 10 Most Significantly Upregulated Proteins in Lacrimal Glands of NOD Mice Compared With BALB/c Mice

Rank^*^	Protein Name	Protein Symbol	Accession ID	Log2 Fold-Change (NOD/BALBc)	Effect Size	Adj. *P* Value^†^
1	Filamin-A	FLNA	Q8BTM8	2.70	3.60	1.26E-67
2	H2B clustered histone 7	H2BC7	P10853	3.10	2.79	1.98E-45
3	Annexin A2	ANXA2	P07356	2.35	2.83	1.60E-42
4	Actin, aortic smooth muscle	ACTA2	P62737	2.53	2.29	4.81E-34
5	Histone H4	H4C1	P62806	3.10	2.89	4.94E-32
6	Vimentin	VIM	P20152	2.49	3.23	1.07E-31
7	Aminopeptidase N	ANPEP	P97449	1.16	1.52	1.27E-29
8	Heterogenous nuclear ribonucleoprotein M	HNRNPM	Q9D0E1	1.80	2.61	4.65E-28
9	Actin, cytoplasmic 2	ACTG1	P63260	1.51	1.87	5.65E-28
10	Plastin-2	LCP1	Q61233	2.81	3.54	4.21E-27

* All significantly differentially expressed proteins were ranked by taking into consideration both their Log2 fold-changes and adjusted *P* values using the following equation: Ranking = SIGN(log2 FC) × −log10 (adj. *P* value).

† *P* values adjusted after correction for multiple comparisons.

**Table 2.** Top 10 Most Significantly Downregulated Proteins in Lacrimal Glands of NOD Mice Compared With BALB/c Mice

Rank^*^	Protein Name	Protein Symbol	Accession ID	Log2 Fold-Change (NOD/BALBc)	Effect Size	Adj. *P* value^†^
1	Valyl-tRNA synthetase	VARS1	Q9Z1Q9	−2.96	−3.19	1.42E-62
2	Odorant-binding protein 1a	OBP1A	Q9D3H2	−3.65	−3.20	7.54E-58
3	Arginase-1	ARG1	Q61176	−4.91	−3.75	2.71E-25
4	Methanethiol oxidase	SELENBP1	P17563	−1.45	−1.55	4.21E-17
5	Carboxypeptidase D	CPD	O89001	−1.36	−1.36	1.97E-12
6	2-Oxoadipate dehydrogenase complex E1	DHTKD1	A2ATU0	−3.78	−2.82	2.03E-12
7	Ras-related protein Rab-3D	RAB3D	P35276	−1.04	−1.81	6.70E-12
8	Eukaryotic translation initiation factor 5	EIF5	P59325	−1.36	−1.52	1.34E-11
9	Mitochondrial dicarboxylate carrier	SLC25A10	Q9QZD8	−1.31	−2.15	2.23E-11
10	Glycerol-3-phosphate dehydrogenase	GPD1	P13707	−1.61	−1.56	3.59E-11

Negative fold-changes and effect sizes indicate that protein expression is downregulated in NOD compared with BALB/c samples.

* All significantly differentially expressed proteins were ranked by taking into consideration both their Log2 fold-changes and adjusted *P* values using the following equation: Ranking = SIGN(log2 FC) × −log10 (Adj. *P* value).

† *P* values adjusted after correction for multiple comparisons.

In our DEA approach, we chose not to impute abundances for missing values. Accordingly, proteins that are predominantly found in one sample group cannot be statistically evaluated by the differential expression analysis algorithm. Instead, we used the differential detection algorithm and resulting z-scores to identify group-specific proteins. A total of 44 proteins were differentially detected between NOD and BALB/c samples. Table 3 shows the top 10 differentially detected/found proteins in LGs from NOD mice and the 4 only proteins predominantly found in BALB/c samples (see Supplementary Table S4 for the complete list of differentially detected proteins).

**Table 3.** Top 10 Differentially Detected/Found Proteins in Either NOD or BALB/c Samples

Rank^*^	Protein Name	Protein Symbol	Accession ID	Log2 Fold-Change (NOD/BALBc)	Effect Size	Adj. *P* Value^†^
In NOD samples						
1	Intercellular adhesion molecule 1	ICAM1	P13597	3.43	10.42	1.23E-22
2	Lymphocyte-specific protein 1	LSP1	P19973	3.43	10.41	1.23E-22
3	Trans-Golgi network protein 2	TGOLN2	Q62314	3.20	9.70	9.40E-20
4	EH domain-containing protein 3	EHD3	Q9QXY6	3.19	9.69	9.40E-20
5	Dihydropyrimidinase like 3	DPYSL3	Q62188	3.13	9.51	4.18E-19
6	Nucleoporin 85	NUP85	Q8R480	3.06	9.30	2.50E-18
7	Septin 6	SEPTIN6	Q9R1T4	3.06	9.28	2.50E-18
8	DNA topoisomerase I	TOP1	Q04750	3.05	9.26	2.50E-18
9	Galectin 3	LGALS3	P16110	3.05	9.26	2.50E-18
10	Dedicator of cytokinesis 2	DOCK2	Q8C3J5	3.01	9.13	7.43E-18
In BALB/c samples						
1	Synaptotagmin like 1	SYTL1	Q99N80	−2.64	−8.01	7.68E-14
2	Beta-1,4-galactosyltransferase 1	B4GALT1	P15535	−2.53	−7.68	8.55E-13
3	Component of oligomeric Golgi complex 8	COG8	Q9JJA2	−2.10	−6.39	5.21E-09
4	APAF1 interacting protein	APIP	Q9WVQ5	−2.07	−6.29	9.53E-09

Negative fold-changes and effect sizes indicate that protein expression is downregulated in NOD compared with BALB/c samples.

* All significantly differentially detected proteins were ranked by taking into consideration both their Log2 fold-changes and adjusted *P* values using the following equation: Ranking = SIGN(log2 FC) × −log10 (Adj. *P* value). Only four proteins were identified as differentially detected in BALB/c samples compared with NOD samples using the differential detection algorithm.

† *P* values adjusted after correction for multiple comparisons.

### Up- and Down-Regulated Pathways Using Gene Set Enrichment Analysis

**Figure 4.**
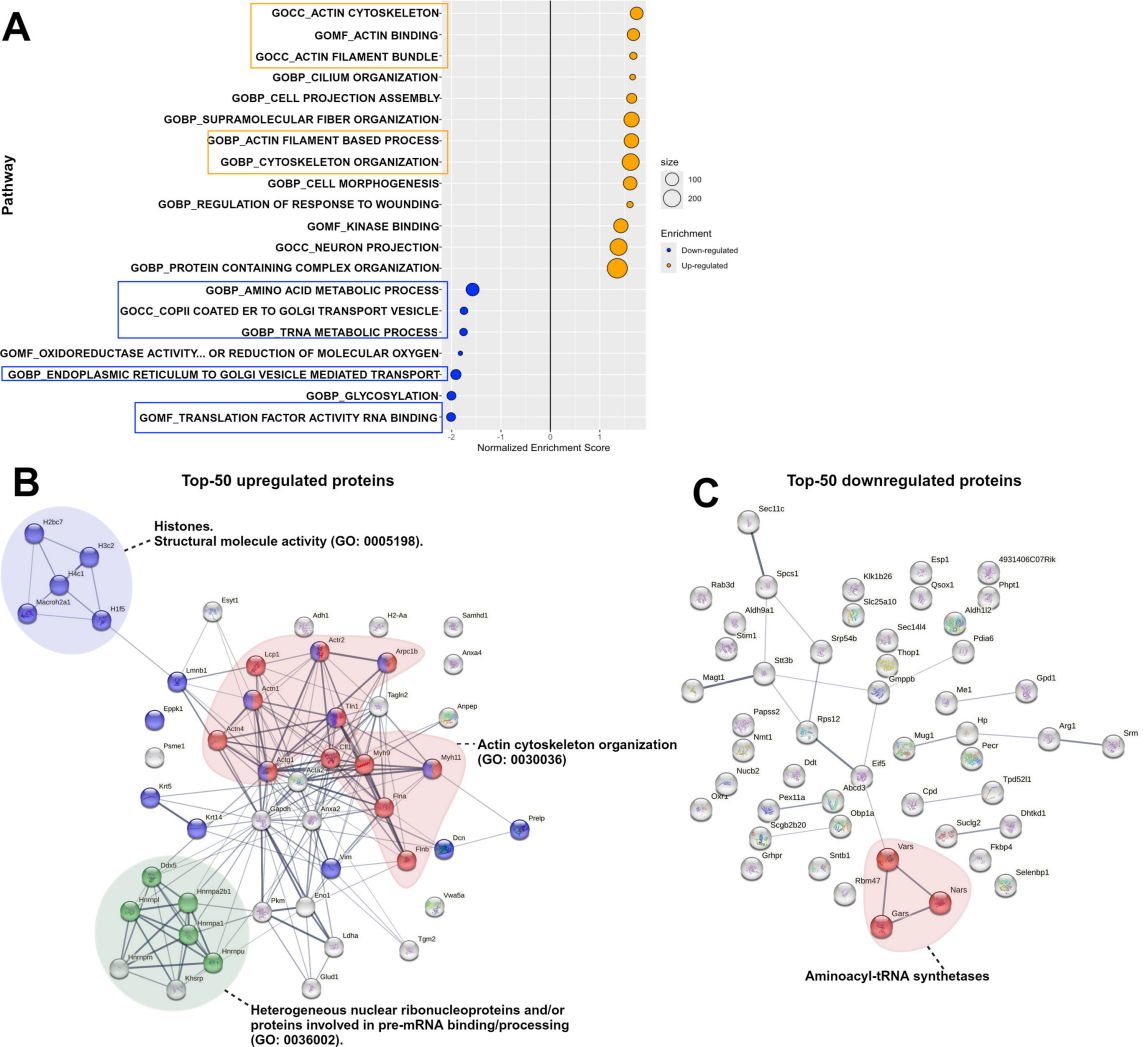
**Regulation of Gene Ontology (GO) terms and protein-protein interactions.** (**A**) All quantified differentially expressed and detected proteins were ranked by their effect sizes and −log_10_ *P* values prior to being subjected to gene set enrichment analysis (GSEA) against the M5 mouse collection of the Molecular Signature Database, which contains expression signatures of three root GO ontologies: biological processes (prefixed with “GOBP”), cellular components (prefixed with “GOCC”), and molecular functions (prefixed with “GOMF”). The top 20 enriched pathways are shown with upregulated (*orange bubbles*) and downregulated (*blue bubbles*) pathways listed on the y-axis while the x-axis represents the normalized enrichment scores. The top 50 upregulated (**B**) and top 50 downregulated (**C**) differentially expressed proteins were inputted into the STRING database to evaluate protein-protein interactions or associations. Interaction network maps were generated with a medium confidence (0.400) and the main interacting clusters observed were color highlighted.

All quantified proteins were ranked based on their effect sizes and adjusted *P* values prior to being subjected to GSEA against the M2, M5, and M8 mouse collections of the Molecular Signatures Database (MSigDB). To elucidate the biological functions of the identified proteins with different expression levels in healthy versus diseased LGs, we analyzed the enriched gene sets against the M5 collection of the MSigDB. The M5 collection is derived from gene ontology (GO) resources and represents GO terms belonging to three root ontologies: biological processes (BPs), cellular components (CCs), and molecular functions (MFs). In our dataset, a total of 62 pathways and another additional 7 pathways from the M5 collection, were upregulated and downregulated, respectively. Multiple pathways related to actin/actin-binding and actin filament components of the cytoskeleton were upregulated among the top 20 enriched gene sets in our analysis (Fig. 4A), suggesting that many of the differentially expressed proteins upregulated in diseased LGs from NOD mice were cytoskeletal proteins. Conversely, amino acid and tRNA metabolic processes were two metabolism-associated pathways downregulated in our analysis against the M5 collection. The largest molecular function downregulated was “translation factor activity RNA binding” whereas a set of pathways related to vesicle mediated transport from the endoplasmic reticulum to the golgi apparatus were also highly downregulated in our dataset (see Fig. 4A).

Additionally, protein-protein interactions were evaluated using the STRING database (https://string-db.org/). The top 50 (for data visualization purposes) up- and downregulated proteins in the dataset were inputted into the database and protein association or interaction network maps were generated with a medium confidence (0.400) criterion. Similar to the GSEA results, proteins involved in the organization of the actin cytoskeleton were the largest interacting cluster observed among the top 50 upregulated proteins, followed by histone proteins or proteins with similar molecular structure activity, and, last, heterogeneous nuclear ribonucleoproteins involved in pre-mRNA binding and RNA metabolism (Fig. 4B). Conversely, very few protein-to-protein interactions were present among the top 50 downregulated protein list, with a small interacting cluster consisting of aminoacyl-tRNA synthetases such as Valine, Glycine, and Asparagine-tRNA ligases (Fig. 4C).

**Figure 5.**
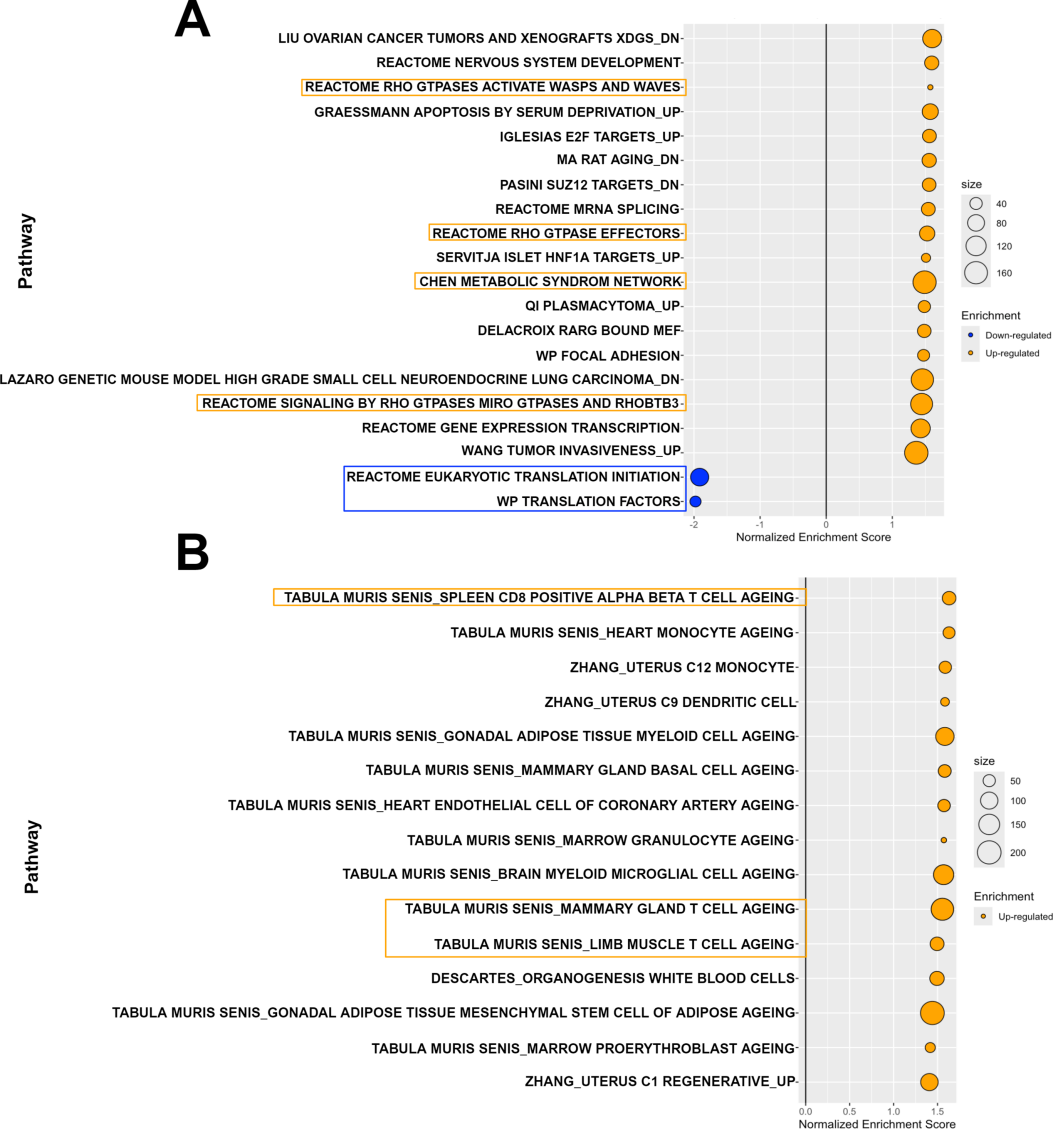
**Regulation of curated canonicals pathways and cell type cluster marker genes.** All quantified differentially expressed and detected proteins were ranked by their effect sizes and −log_10_ *P* values prior to being subjected to gene set enrichment analysis (GSEA) against the M2 and M8 mouse collections of the Molecular Signature Database. (**A**) Bubble plot showing the top 20 enriched pathways in our dataset from the M2 collection which contains curated gene sets divided into expression signatures of genetic and chemical perturbations, and canonical pathways. (**B**) All 15 enriched pathways from the M8 collection which contains cluster marker genes for cell types identified in single-cell sequencing studies of mouse tissue. Enrichments are [NOD]/[BALB/c] and more gene sets were upregulated than downregulated.

The M2 collection contains gene sets from curated online pathways databases and the biomedical literature, which are divided into chemical and genetic perturbations, as well as canonical pathways.^30^ GSEA against the M2 collection revealed upregulation of 19 pathways in LGs from NOD mice and 2 downregulated pathways. Among the top 20 enriched pathways presented in Figure 5A, gene sets associated with translation initiation and translation factors were the 2 downregulated canonical pathways. Meanwhile, the “Chen metabolic syndrome network” pathway (comprised of genes forming the macrophage-enriched metabolic network related to metabolic syndrome traits), and several Rho GTPases signaling pathways, were significantly upregulated in diseased LGs from NOD mice when compared with BALB/c mice.

Last, GSEA against the M8 collection facilitated the identification of up- or downregulated cell type signatures among the differentially expressed and detected proteins in our dataset. The M8 collection of the MSigDB contains cluster marker genes for cell types that have been identified in different single-cell sequencing studies of mouse tissue. Fifteen total pathways were upregulated, and none significantly downregulated, as shown in Figure 5B. Not surprisingly, several cluster marker gene sets for cell types associated with T-cell aging were significantly upregulated in the chronically inflamed LGs of NOD mice. Most of the remaining significantly upregulated pathways were associated with immune-system-related cell types, such as monocytes and myeloid cells aging.

Overall, our GSEA shows that more gene sets or pathways were found to be upregulated than downregulated in chronically inflamed LGs of diseased NOD mice, suggesting that significant alterations are taking place in the LG proteome after the development of dacryoadenitis.

### Validation of LC-MS/MS Results Using Immunofluorescence Staining

**Figure 6.**
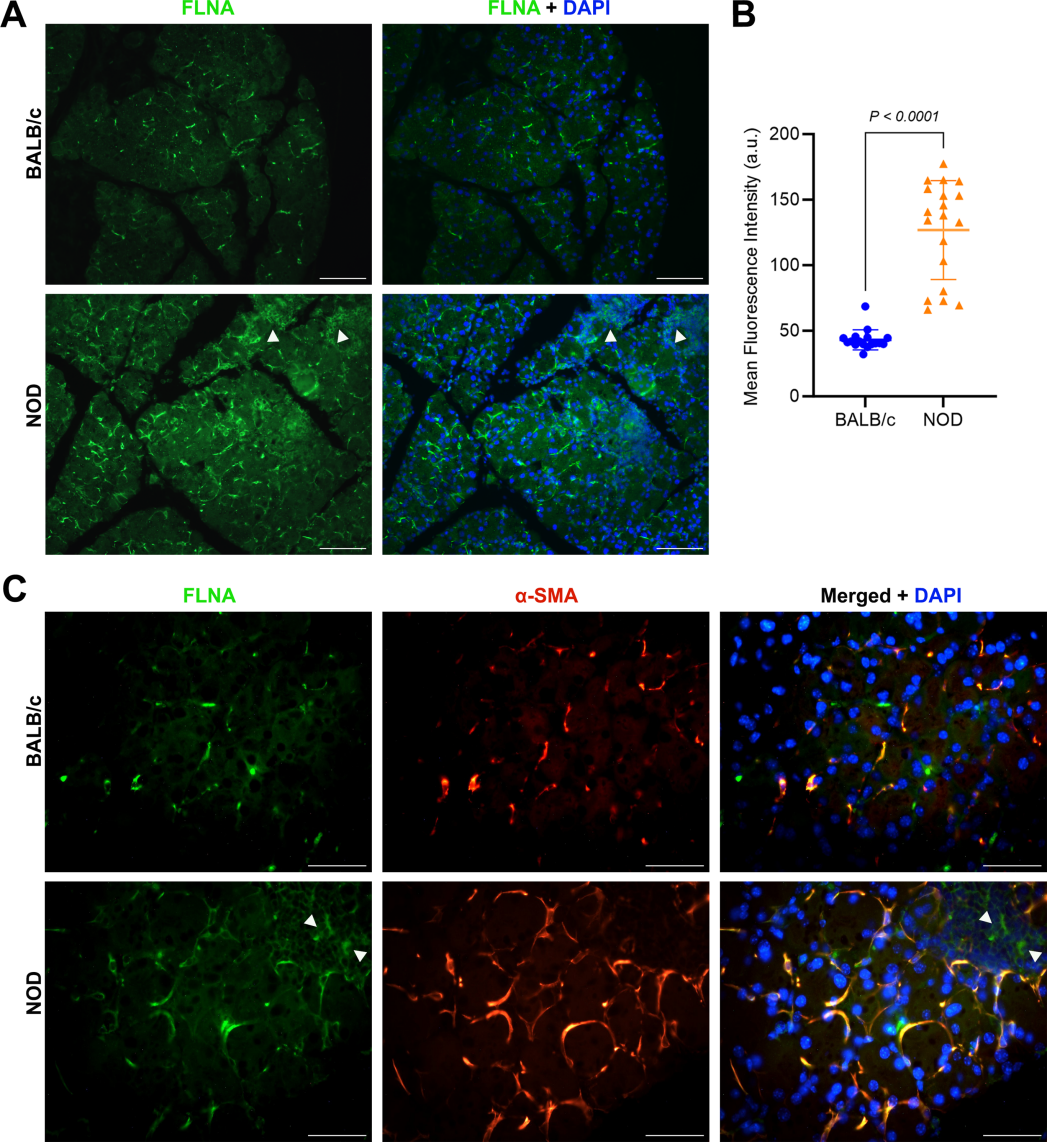
**Filamin-A upregulated expression and co-localization with alpha-smooth muscle actin.** (**A**) Representative immunofluorescence photomicrographs of filamin-A (FLNA) staining in BALB/c (*top*) versus NOD (*bottom*) LG tissue sections. *Arrow heads* depict positive FLNA staining present in tissue-infiltrated lymphocytes. *Scale bars* = 100 µm. Primary antibody was omitted for negative control sections (see Supplementary Fig. S2). (**B**) FLNA staining was statistically significantly higher in NOD samples when compared with BALB/c samples. Data plot is presented as means ± SD, *n* = 17 to 19 from 4 different mice for each strain. (**C**) Representative immunofluorescence photomicrographs of LG tissue sections from control (*top*) and diseased (*bottom*) animals double-stained for FLNA and alpha-smooth muscle actin (α-SMA). *Arrow heads* depict positive FLNA staining present in the tissue-infiltrated lymphocytes. *Scale bars* = 50 µm. The merged channels (FITC + TRITC) images (*right*) show strong FLNA co-localization with α-SMA expressed in the myoepithelial cells of the LG.

To validate the LC-MS/MS results, selected proteins with significant differential expression or differentially detected in healthy controls versus diseased mice were subjected to spatial expression analysis using immunofluorescence staining*.* One of the most significantly differentially upregulated proteins (2.70 positive fold-change, adjusted *P* value = 1.26E-67) in LGs from diseased animals compared with controls was Filamin-A (FLNA). As shown in Figures 6A and 6B, immunofluorescence staining confirmed the overexpression of FLNA in diseased LGs. Moreover, and because the staining pattern of FLNA was very similar to that of alpha-smooth muscle actin (α-SMA), an important contractile protein expressed by myoepithelial cells (MECs) of the lacrimal and other exocrine glands, LG tissue sections were double stained with anti FLNA and anti α-SMA antibodies. Double staining immunofluorescence showed that FLNA was very strongly co-localized with α-SMA (Fig. 6C), suggesting that this actin-binding protein is greatly upregulated in the MECs of inflamed LGs from the diseased animals. Additionally, a positive FLNA staining signal was observed in some areas where infiltrated lymphocytes were localized within the gland (see Fig. 6C).

**Figure 7.**
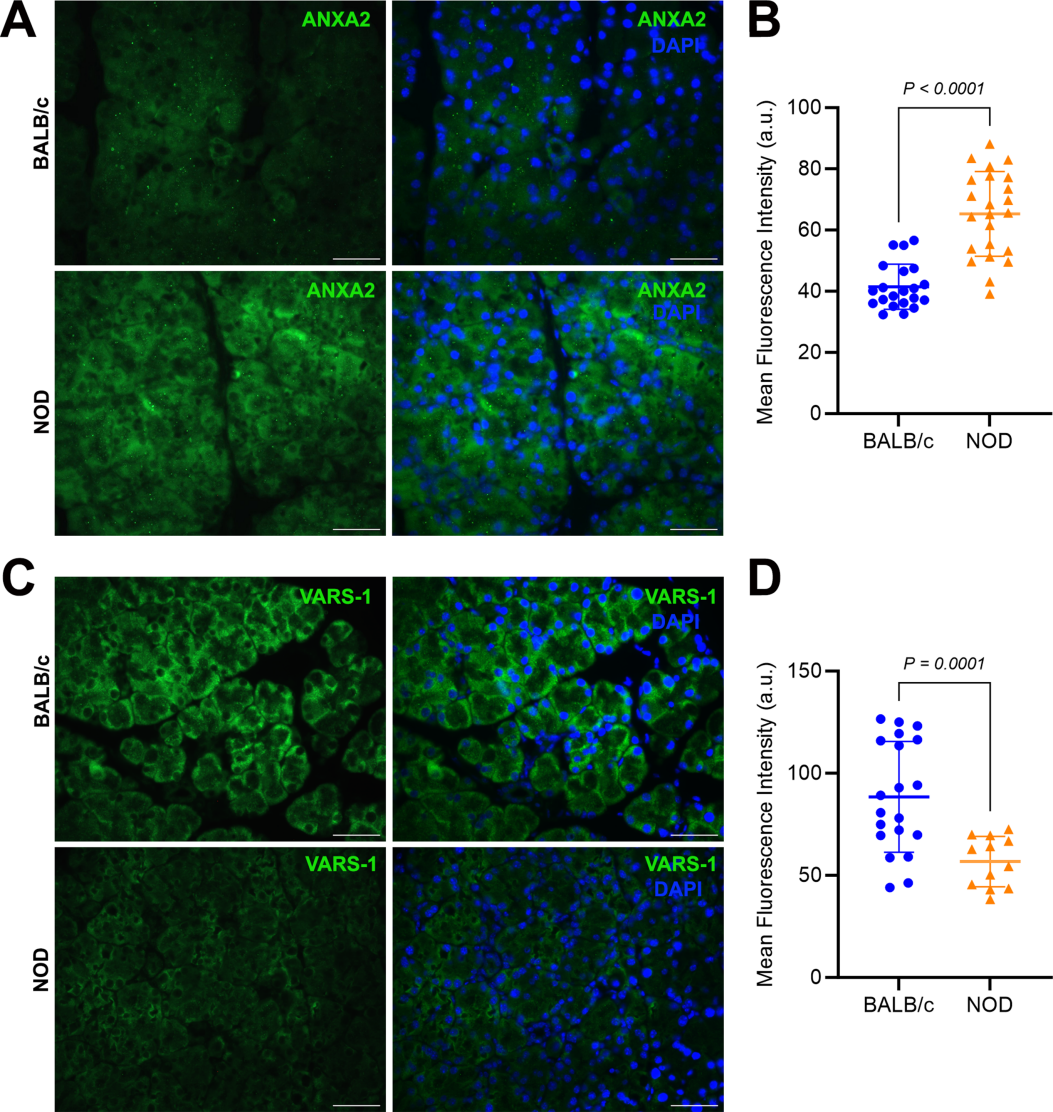
**Immunohistochemical confirmation of additional selected differentially expressed proteins.** (**A**) Representative photomicrographs of annexin A2 (ANXA2) staining in BALB/c (*top*) versus NOD (*bottom*) LG tissue sections. *Scale bars* = 50 µm. Primary antibody was omitted for negative control sections (see Supplementary Fig. S2). (**B**) ANXA2 staining was statistically significantly higher in NOD samples when compared with BALB/c samples; *n* = 21 to 23 from 3 different mice per group. (**C**) Representative photomicrographs of valyl-tRNA synthetase (VARS-1) staining in BALB/c (*top*) versus NOD (*bottom*) LG tissue sections. *Scale bar* = 50 µm. (**D**) VARS-1 staining was statistically significantly lower in NOD samples when compared with BALB/c samples; *n* = 12 to 20 from 3 different mice per group. Data plots are presented as means ± SD.

The upregulated expression of Annexin A2 (ANXA2; Figs. 7A, 7B) and downregulated levels of Valyl-tRNA synthetase (VARS1; Figs. 7C, 7D) in diseased LGs was also analyzed and confirmed by spatial immunofluorescence staining. In addition, LG sections from healthy, young NOD and BALB/c (3 weeks old) mice were also stained for VARS1 and the fluorescence staining intensity semi-quantified. In contrast to the results from stained LG sections from diseased animals and their age-matched controls, the measured VARS1 staining intensity was not lower in these very young NOD mice when compared with age-matched BALB/c controls (83.70 ± 10.48 in BALB/c versus 104.4 ± 21.37 in NOD; Supplementary Fig. S3).

**Figure 8.**
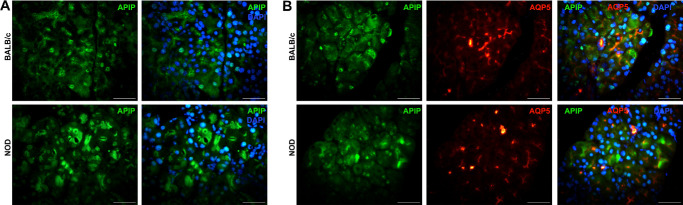
**Immunohistochemical staining of APAF1 interacting protein (APIP)**. (**A**) Representative immunofluorescence photomicrographs of APIP staining in BALB/c (*top*) versus NOD (*bottom*) LG tissue sections. (**B**) Representative immunofluorescence photomicrographs of LG tissue sections from control (*top*) and diseased (*bottom*) animals double-stained for APIP and Aquaporin-5 (AQP5). All *scale bars* = 50 µm.

APAF1 interacting protein (APIP) was one of the four proteins significantly differentially detected in BALB/c samples, and, thus, downregulated (fold change = –2.07; effect size = –6.29; adjusted *P* value = 9.53E-09) in LGs from diseased NOD mice. APIP was expressed both at the nuclear and cytoplasmic level in the LG acini regions from both the diseased and healthy control animals (Fig. 8A). However, there was no significant difference between the measured fluorescence intensity of APIP-stained LG sections from control versus diseased mice (data not shown). Because APIP seemed to be expressed at the apical portion of the acini, additional LG sections were double-stained with APIP and aquaporin-5 (AQP5) – a water channel protein that is known to be expressed at the apical region of secretory cells of lacrimal and salivary glands. Based on the double staining results, APIP was not precisely co-localized with AQP5 in acinar and ductal cells, however, the staining localization of APIP did seem to be near that of AQP5 present in the acini (Fig. 8B). Additionally, no fluorescence staining intensity difference was observed between young and healthy (3 weeks old) NOD and age-matched BALB/c LG sections stained for APIP (97.08 ± 32.41 in BALB/c versus 92.13 ± 22.24 in NOD; see Supplementary Fig. S3).

## Discussion

We have previously reported on how focal lymphocytic infiltration of the LG, as well as the presence of autoantibodies and proinflammatory cytokines, significantly impair LG function and secretion.^4^^,^^8^^,^^9^^,^^31^ In this study, we present a foundational description and characterization of the LG proteome and the overall changes to its protein expression patterns induced by the reported chronic inflammation pathology. Nanoflow LC-MS/MS was used to compare LG protein extracts from BALB/c (wild type) and NOD (Sjögren's disease murine model) mice. Differential expression analysis was utilized to analyze and interpret protein expression differences between the two sample groups. A total of 1750 proteins were identified and analyzed after global peptide filtering, resulting in 580 proteins with statistically significant expression differences between healthy and diseased LG samples. Moreover, GSEA of ranked differentially expressed proteins revealed more upregulated pathways and fewer downregulated ones in NOD samples. Several proteins from the top 10 most significantly upregulated list were cytoskeleton proteins, subdivided mainly into actin/actin-binding proteins such as FLNA, Plastin-2, and Actin, aortic smooth muscle, or intermediate filament proteins like Vimentin (VIM). The upregulation of proteins involved in the organization of the cytoskeleton and actin filament-based processes in LGs from the diseased animals was further corroborated by the GSEA and protein-protein interaction network results – where such pathways were found to be some of the most upregulated gene ontology terms among all the protein sets analyzed.

The increased expression of VIM has been previously reported in LGs of ocular graft versus host disease and IgG4 dacryoadenitis,^32^ however, and to our knowledge, no studies have been published regarding the overexpression of FLNA in chronically inflamed LGs from patients or animal models of Sjogren's and dry eye disease. FLNA is a homodimer actin-binding protein (approximately 280 kDa) involved in the formation of the cytoskeleton and has been reported to anchor various proteins in the cytoskeleton as well as to regulate cell adhesion and migration.^33^ Additionally, FLNA has been shown to be involved in signal transduction, cell proliferation and differentiation, tumor resistance, and genetic diseases by its binding to interacting proteins.^33^ FLNA plays a crucial role in determining the shape and movement of cells by guiding the formation of dynamic actin stress fibers and by acting as a scaffold for signaling proteins, such as tyrosine kinases, GTPases, or phosphatases, and adhesion receptors such as integrins.^33^^,^^34^ Positive FLNA staining was observed in areas of lymphocytic infiltration present in the diseased LGs. Among the three filamin isoforms (FLNA, FLNB, and FLNC), FLNA has been identified to be predominantly expressed in macrophages^35^^,^^36^ and in T-cells, with several studies indicating that FLNA plays a crucial role in T-cell activation.^37^^–^^39^ Because FLNA interacts with adhesion receptors such as integrins, which are necessary for the trafficking of T-cells from the bloodstream into various tissues, FLNA is needed for the formation of strong integrin-ligand bonds,^35^ and, thus, possibly explains one of the reasons why a significant upregulation of FLNA was observed in chronically inflamed LGs from the diseased mice when compared with healthy glands from control animals. Studies of mice with FLNA-deficient macrophages to target FLNA, and its cleavage with calpain, have been shown to reduce atherosclerosis in these animals, with FLNA-deficient macrophages migrating and proliferating poorly as well as secreting lower levels of inflammatory cytokines, IL-6, and IL-12.^35^^,^^36^ Similarly, FLNA has been reported to be involved in the regulation of immune cells and fibroblasts,^40^ with observed decreased mRNA levels of inflammatory cytokines and chemokines, and suppression of STAT3 signaling in FLNA-downregulated macrophages, or increased levels of metalloproteinases and pro-apoptotic proteins in FLNA knockdown immortalized human hepatic stellate cells.^40^

Additionally, in the present study, FLNA staining was strongly co-localized with α-SMA in the MECs of the LG. Different studies have shown that an increased expression of FLNA enhances the epithelial-mesenchymal transition (EMT) in cancer cells.^41^^–^^43^ We have previously shown that EMT plays a key role in LG repair and regeneration after experimentally induced injury.^44^ MECs play a critical role in secreting and maintaining the extracellular matrix (ECM) and basement membrane^45^ of the LG, thus, such upregulation of FLNA expression in MECs could potentially disrupt the normal rate at which these cells undergo EMT and subsequent mesenchymal epithelial transition (MET) and/or diminish their ability to maintain a healthy ECM and stem/progenitor cell niche needed for proper LG repair and regeneration.

Given the significant overexpression of VIM in the chronically inflamed LGs presented in this study, and how permanent ECM remodeling has been suggested to impair LG regeneration after substantial injury – with LG inflammation reported to deregulate important ECM components and alter the molecular signature of epithelial stem/progenitor cells,^46^ together with the fact that VIM intermediate filaments have the ability to regulate collagen deposition and matrix remodeling,^47^ further studies investigating the role of VIM in LG ECM remodeling could help better understand the mechanisms involved in impaired LG regeneration as occurs in chronically inflamed LGs. Similarly, the studies described above regarding the role of FLNA in inflammation, suggest that the design and screening of small molecules to inhibit the cleavage or expression of FLNA, or the design of inhibitors which specifically block the interactions of FLNA with its partners, could contribute to the reduction of inflammation, posing FLNA, VIM, and the other upregulated cytoskeletal proteins described in this study, as potential biomarkers or therapeutic targets for inflammatory diseases like Sjogren's disease.

Another class of proteins highly upregulated in the present study of chronically inflamed LGs is annexins, with all annexin A1, A2, A3, A4, and A11, found to be significantly upregulated in diseased LGs compared with healthy controls. Annexins are calcium and phospholipid binding proteins with rapid translocation in cells from the cytosol to the intracellular and plasma membrane that undergo post-translational modifications enabling them to interact with a wide range of cellular components, and thereby, increasing their interactions with and regulations of cellular products and functions involved in inflammation and immune activities, vesicle trafficking, cell growth, cell division and differentiation, and programmed cell death.^48^ The most significantly upregulated annexin type in our proteomics dataset was annexin A2 (ANXA2), which has been previously reported to be the strongest upregulated annexin gene in the LG based on temporal genome-wide RNA transcriptome analyses of ex vivo salivary and LGs from a Sjögren's disease mouse model.^48^ ANXA2 is expressed in multiple tissues and cell types, including epithelial and endothelium, macrophages, monocytes, trophoblasts, and dendritic and tumor cells.^48^^,^^49^ The over- and under-expression of annexins have been identified in several auto-immune diseases, such as rheumatoid arthritis, Sjögren's disease, and systemic lupus erythematosus,^48^ supporting the findings presented in this study at the protein level. For instance, a study by Cui et al., found ANXA2 to be overexpressed in the salivary glands of patients with primary Sjögren's disease/mucosal-associated lymphoid tissue (MALT) lymphoma, suggesting that ANXA2 possibly contributes to the pathophysiology of Sjögren's disease, and could represent a potential biomarker of disease progression and lymphoproliferation in patients with the disease.^48^^,^^50^ Last, ANXA2 has been shown to promote the EMT by regulating multiple signaling pathways,^51^^–^^55^ and its expression has been reported to promote Toll-like receptor 4 (TLR4) translocation into the early endosomal membrane to activate the TRAM-TRIF endosomal signal, leading to the release of anti-inflammatory cytokines.^56^ Nonetheless, not much has been reported on how exactly annexins contribute to the pathophysiology of Sjögren's disease, and thus, further studies are needed.

It is widely known that histone modifications regulate transcription mechanisms, such as transcription factor binding and gene expression.^57^ As shown in the protein-protein interaction network diagram, several histone proteins, such as H2B clustered histone 7 (H2BC7) and histone H4 (H4C1), to name a couple, were significantly overexpressed in LGs from NOD mice compared with BALB/c samples. Previous studies have suggested that phosphorylation of histone H2B at different serine locations is associated with resting B-cell death^58^ and regulation of cell survival in response to stress.^59^ On the other hand, externalized histone H4 has been reported to mediate membrane lysis of smooth muscle cells and induce arterial tissue damage and inflammation, thus, mediating chronic inflammation by inducing lytic cell death.^60^ Interestingly, inhibition of histone H4 with a histone inhibitory peptide, prevented histone H4 from interacting with and altering the cellular membrane, greatly reducing smooth muscle cell death.^60^ Given the overexpression of these several histone proteins in diseased LGs compared with healthy controls as identified in this study, the regulation of these histone proteins via the use of peptide-based histone inhibitors could have potential therapeutic uses for the prevention of tissue damage and chronic inflammation.

Enzymes involved in important catalytic and metabolic biological functions were some of the most significantly downregulated proteins in our dataset. As shown by the protein-protein interaction network analysis, aminoacyl-tRNA synthetases such as Valine-tRNA synthetase (VARS1), Glycine-tRNA synthetase (GARS), and Asparagine-tRNA synthetase (NARS) were significantly downregulated in the diseased LGs compared with healthy controls. VARS1, a member of 20 essential enzymes categorized as aminoacyl-tRNA synthetases (ARSs) and whose main function is to ligate valines to their corresponding tRNAs during protein synthesis,^61^^,^^62^ was the most significantly downregulated ARS enzyme in our dataset. Nothing has been previously reported regarding the regulation or involvement of VARS1 in dry eye disease, however, and in accordance with our findings, weighed gene co-expression network analysis of data from patients with melanoma obtained from The Cancer Genome Atlas, identified VARS1 expression to be inversely correlated with immune-related signaling pathways and the expression of several immune checkpoint genes.^62^ More precisely, high VARS1 expression correlated with small CD8 T-cell infiltration while immune-related pathways such as T-cell or B-cell receptor signaling pathways were enriched in patients with low VARS1 expression.^62^ Thus, aligning with our observations of downregulated VARS1 expression in LGs infiltrated with lymphocytes. Furthermore, recent advances in genomic, proteomic, and functionomic have demonstrated interesting mutations, altered expression, and interactions in human ARSs, associating them with diseases and biological functions beyond their catalytic roles in protein synthesis.^61^ ARSs allow for drug development through multiple routes by targeting their catalytic sites, targeting disease-associated protein-protein interactions, or by targeting secreted forms of ARSs – which have been reported to control cellular processes such as immunity, angiogenesis, tissue regeneration, and metabolism. Multiple ARS-derived biomarkers and therapeutics are under development as potential indicators or treatment modalities for diseases such as malaria, different cancers, rheumatoid arthritis, fibrosis, bacterial infections, and several more.^61^ Given the significant downregulation of VARS1 in chronically inflamed LGs as identified in this study, the potential involvement of ARSs in other functions beyond protein synthesis, and the ability to target and regulate these synthetases with therapeutics via multiple routes, future studies examining a potential role of VARS1 in immune-related signaling pathways during chronic inflammation of the LG could provide key insights into whether or not VARS1 could be positioned as a novel biomarker of disease progression and lymphoproliferation, and a therapeutic target for autoimmune or inflammation-related diseases such as Sjögren's disease.

Last, the MS-DAP data analysis pipeline used in this study provides extensive quality control measures, state-of-the-art algorithms for differential testing and visualization/reporting of the results, as well as custom functions for normalization or differential expression analysis, all of which support a robust interpretation of MS-based proteomics experiments. Nevertheless, we would like to note potential limitations of this study. First, we recognize that the small sample size used in this study does not allow for subcategorization of dry eye and Sjögren's disease animal models according to the severity and progression of the disease. The old (>30 week old) NOD mice used in this study show severe lymphocytic infiltration and dry eye, however, the early and developing stages onset of both LG hyposecretion and dacryoadenitis starts as early as 6 to 10 weeks of age in NOD mice,^63^^,^^64^ and, therefore, future proteomics-based studies using animal models of different ages are needed to detect proteins and events altered at different disease stages and progression. Second, even though BALB/c mice have been the mainstay wild type, control strain used for the investigation of dry eye and Sjögren's disease with NOD mice as the animal model, some concerns have been raised regarding the potential introduction of selection bias in studies using BALB/c mice – a white coated mouse strain that is unrelated to the diseased NOD strain – as controls. Although there is not an ideal control for the NOD strain as used in Sjögren's disease studies, young and healthy NOD mice could be a potential option. However, and because these mice develop immune infiltration in their LGs as such an early age, potential changes in the genome or proteome could then be a result of the large age-gap between healthy and diseased NOD mice. Third, and as with any proteomic profiling study, absolute conclusions cannot be made regarding causative links or correlation between the identified differentially expressed proteins and, in this case, dry eye disease. Future studies are needed to investigate and draw conclusive correlations between candidate protein-based biomarkers and the disease.

In conclusion, the MS-based proteomics analysis presented in this study revealed over 500 significantly differentially expressed proteins in healthy versus chronically inflamed LGs from a well-established animal model of dry eye and human primary Sjögren's disease, suggesting that chronic inflammation leads to significant alterations in the LG proteome. Using GSEA, several cytoskeleton organization and metabolic pathways were identified to be up- and downregulated, respectively, in diseased LGs, suggesting that the expression of many cytoskeletal proteins and metabolic enzymes are being altered in chronically inflamed LGs. These findings suggest that in addition to inflammatory factors, the regulation of many non-inflammatory agents also play a role in tear hyposecretion as occurs in Sjögren's disease. Further investigations are needed to provide insights into the potential clinical relevance of the highlighted protein-based biomarkers.

## Supplementary Material

Supplement 1

Supplement 2

Supplement 3

Supplement 4
