# Supplementary material for: Quantitative Changes in the Proteome of Chronically Inflamed Lacrimal Glands From a Sjögren's Disease Animal Model
Source: Invest Ophthalmol Vis Sci. 2025 Apr 17;66(4):44. doi: 10.1167/iovs.66.4.44 (PMC12013672; doi:10.1167/iovs.66.4.44)
Supplement: Supplement 1 [file iovs-66-4-44_s001.pdf]

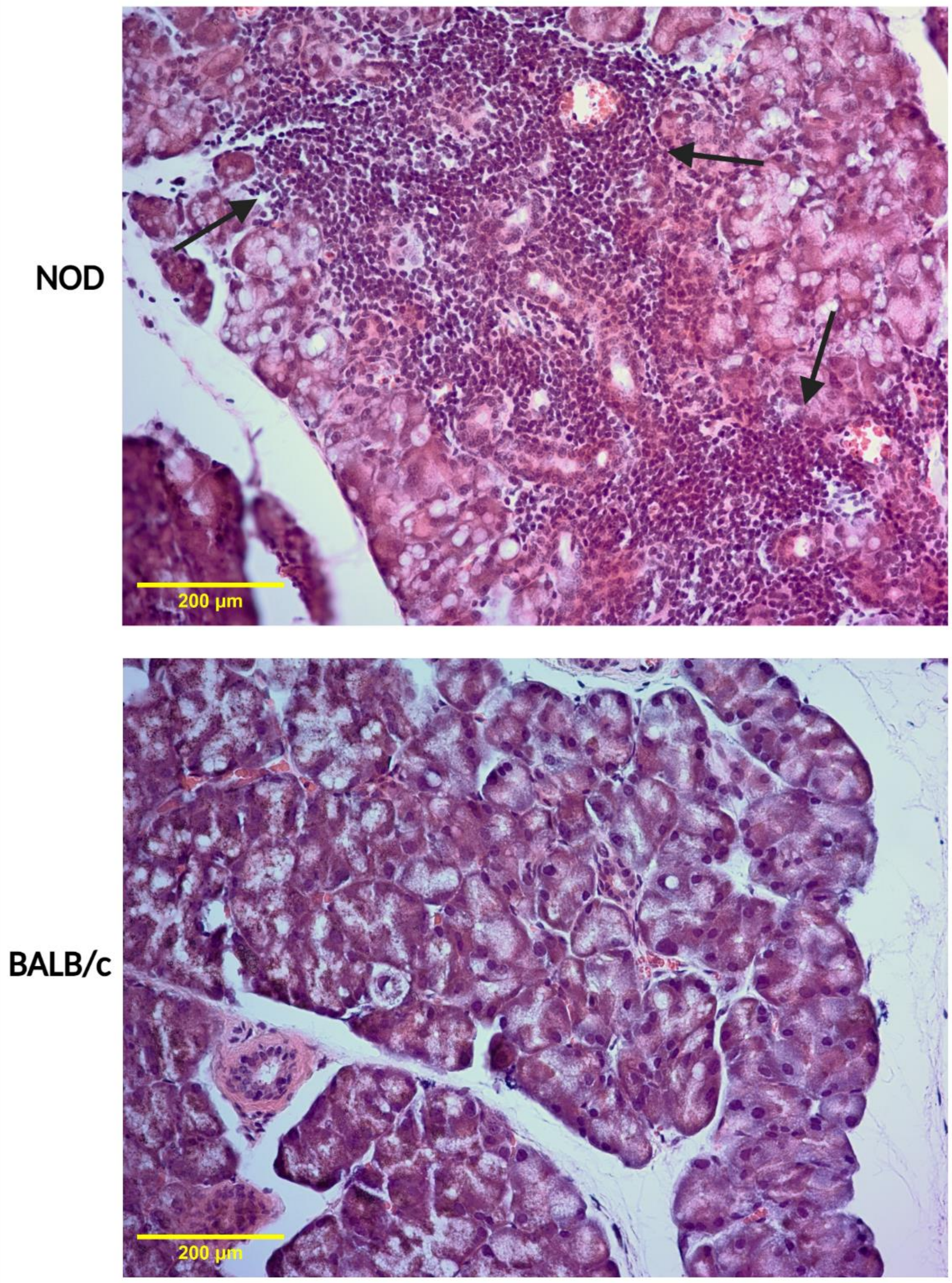

Supplementary Fig. S1. Representative H&E staining photomicrographs of formalin-fixed paraffin-embedded lacrimal glands from NOD and BALB/c mice. Large lymphocytic infiltration (clusters of purplish-blue cells indicated by black arrows) is present in diseased lacrimal glands from NOD mice (top) which is absent in control glands from BALB/c mice (bottom).

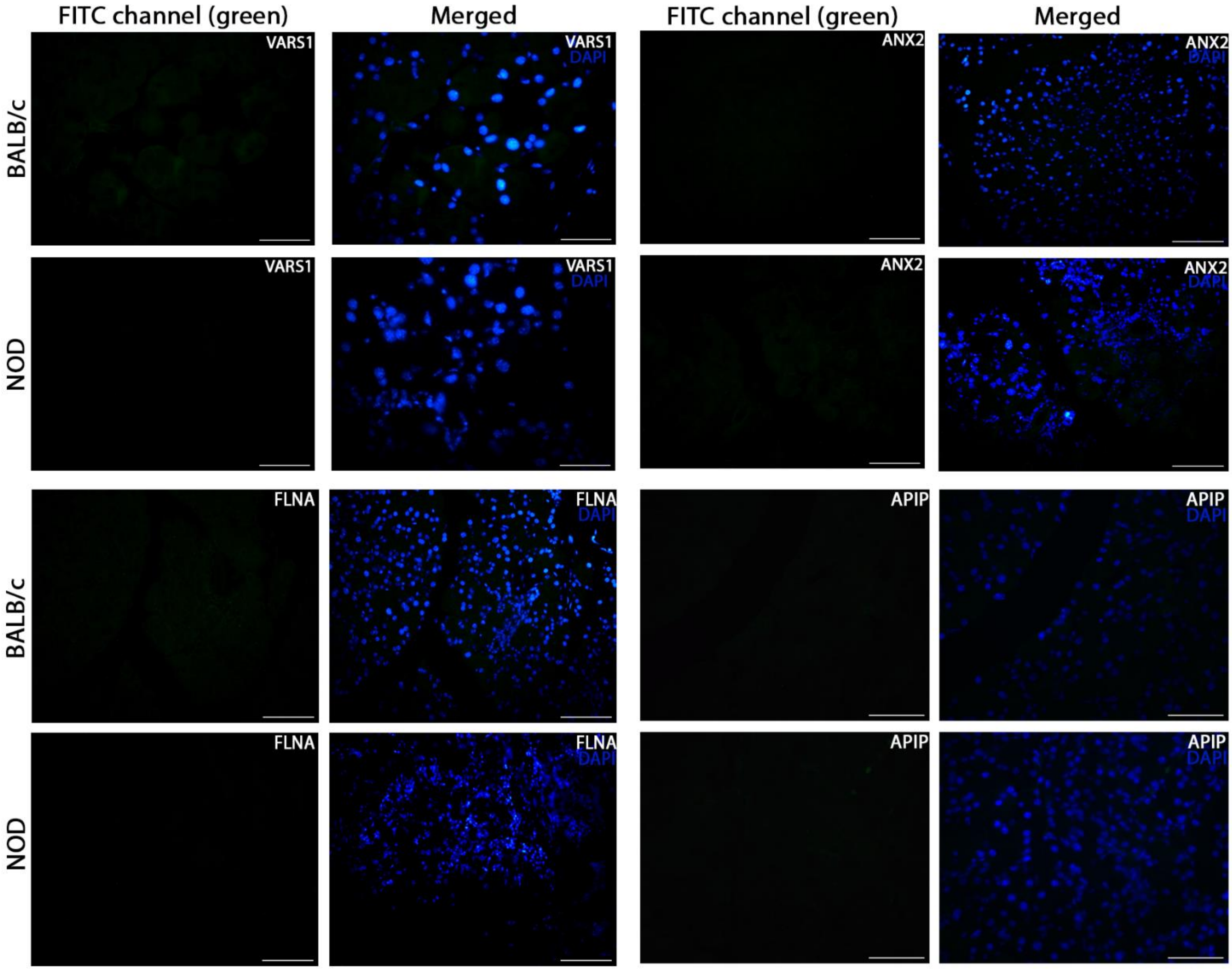

Supplementary Fig. S2. Representative photomicrographs of negative control sections for which the primary antibody was omitted during immunofluorescence staining. No staining (green signal) was observed for tissue sections probed only with the secondary, Alexa Fluor 488 conjugated, antibody. Scale bars = 50 µm, except for FLNA images, which are 100 µm.

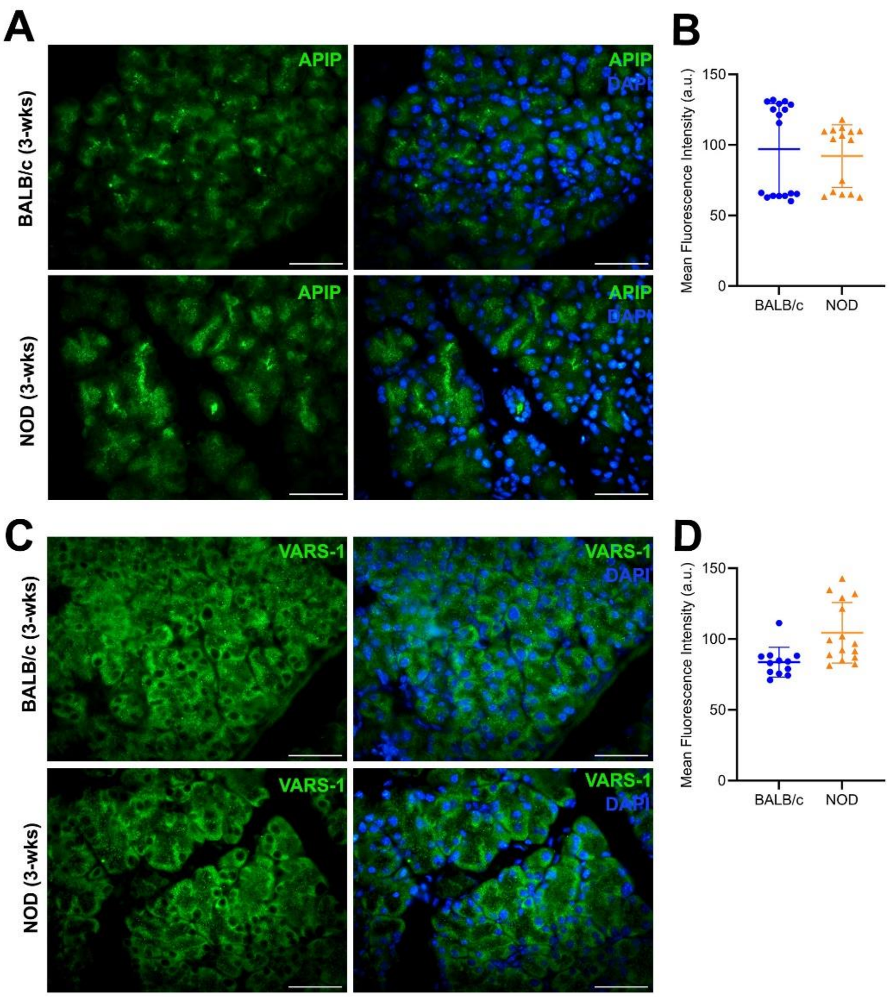

Supplementary Fig. S3. (**A**) Representative photomicrographs of APAF1 interacting protein (APIP) in young (3-weeks-old) BALB/c (top) vs NOD (bottom) LG tissue sections. Scale bars = 50 µm. (**B**) There was no difference in fluorescence intensity between stained young BALB/c and NOD LG sections. n = 15-17 images from two separate animals from each strain. (**C**) Representative photomicrographs of Valyl-tRNA synthetase (VARS1) in young (3-weeks-old) BALB/c (top) vs NOD (bottom) LG tissue sections. Scale bars = 50 µm. (**D**) Contrary to what was observed in old, diseased NOD and their sex- and age-matched BALB/c control LG sections, VARS1 immunofluorescence intensity was not down in the diseased NOD LGs compared to their wild-type controls. n = 12-15 images from two separate animals from each strain.

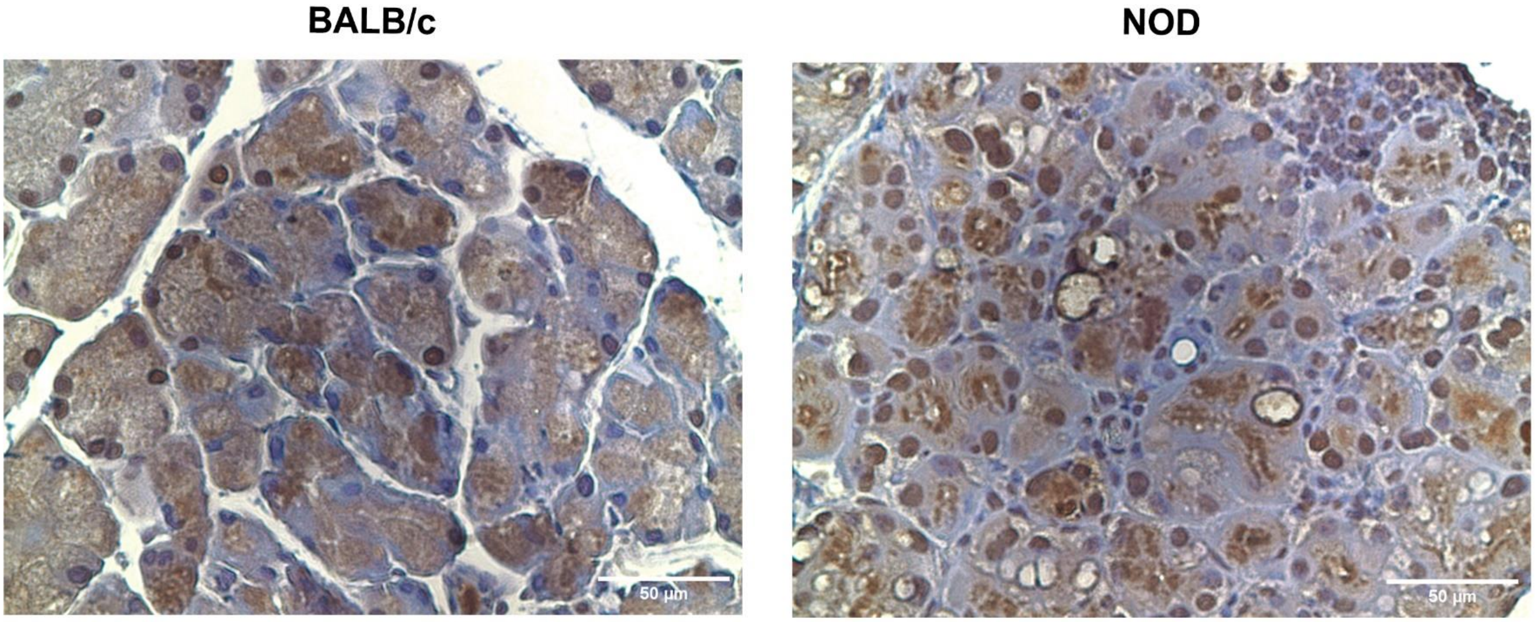

Supplementary Fig. S4. Representative photomicrographs of APAF1 interacting protein (APIP) in the diseased lacrimal gland from NOD mice and age matched BALB/c control. Similar APIP expression was observed at both the nuclear and cytoplasmic level in both strains.

Supplementary Table S1. List of antibodies used for immunofluorescence staining experiments.

Target	Host Species/Conjugate	Vendor	Dilution
Primary Antibodies:			
ANXA2	Rabbit (IgG)	GeneTex (Cat#GTX101902)	1:100
FLNA	Rabbit (IgG)	GeneTex (Cat#GTX112939)	1:200
VARS1	Rabbit (IgG)	GeneTex (Cat#GTX115015)	1:200
APIP	Rabbit (IgG)	Abclonal (Cat#A7102)	1:150
AQP5	Rabbit (IgG)	Proteintech (Cat#20334-1-AP)	1:100
α-SMA	Rabbit (IgG)	abcam (Cat#ab5694)	1:100
Secondary Antibody			
Rabbit (IgG)	Donkey/AlexaFluor 488	abcam (Cat#ab150073)	1:1000
